# Editorial: Women in science: Health economics 2021

**DOI:** 10.3389/fpubh.2022.942087

**Published:** 2022-08-12

**Authors:** Tarang Sharma, Susmita Chatterjee

**Affiliations:** ^1^Evidence to Policy, Hørsholm, Denmark; ^2^Research Department, George Institute for Global Health, New Delhi, India; ^3^Faculty of Medicine, University of New South Wales, Kensington, NSW, Australia; ^4^Prasanna School of Public Health, Manipal Academy of Higher Education, Manipal, India

**Keywords:** women in STEM, health economics, health financing, health policy, women's health

It has been estimated that women spend a disproportionate amount of their time undertaking the world's unpaid work, about 11 billion hours a day and globally women undertake three times more care and domestic work than men. This disparity is more aggravated in low- and middle-income countries (LMICs). The double burden of both unpaid and paid work also has a more detrimental effect on women with studies from USA and Sweden showing links with increased depression in women (1).

This is also coupled with a huge disparity in gender participation the fields of science, technology, engineering, and mathematics, collectively known as STEM. According to the UNESCO Institute for Statistics (UIS) data in 2016, less than 30% of researchers in STEM are women. Furthermore, many studies have shown that women that do pursue STEM fields, publish less, are paid less for their research, and do not progress as far in their careers, as their male counterparts (2). A study from the United States of America (USA) showed that though in 2015, women were half of the college-educated U.S. workforce in science and engineering, they only made up 28% of the workforce in these professions. Another study from the United Kingdom showed a similar figure of only 23% of the scientific workforce being women (3).

It is also widely accepted that women are highly underrepresented in the field of economics, despite many having a huge impact on the field (4, 5). The field of health economics therefore suffers two-fold as it cohabitates both STEM and economics fields together. This special collection series for 2021 of “Women in Science: Health Economics,” hopes to increase the visibility of women in science, specifically in health economics which as other STEM fields seems to be dominated by men receiving the awards and recognition. In this collection, either the first, last or both authors are female health economists, and this showcases five excellent examples of women working in STEM.

The first contribution by Boruzs et al., investigated the possible differences in the beliefs regarding the necessity as well as concerns around lipid-lowering drugs among the Visegrad Group countries (Poland, the Czech Republic, Slovakia, and Hungary). They found that the Hungarian respondents perceived the lowest necessity of these drugs, followed by the Czech and Slovak respondents with the Polish respondents believing the highest necessity of these drugs. However, fears and concerns around these drugs did not differ amongst these countries (Boruzs et al.).

The next contribution in this series by Lin et al., investigated health of women with and without dysmenorrhea, regarding stroke and the related medical care costs using the National Health Insurance Research Database in China. Using data from 1997 to 2013 for women between 15 and 44 years, 66,048 women with dysmenorrhea and 66,048 women without dysmenorrhea, the authors showed that women with dysmenorrhea had a higher stroke risk (HR = 1.26, 95% CI = 1.11–1.42). They also found that the dysmenorrhea cohort had a higher portion of transient cerebral ischemia stroke, which however was associated with least cost of care (US$157 ± 254) (Lin et al.).

The third contribution by Tuczyńska, Matthews-Kozanecka, et al., conducted a targeted scoping literature review of the accessibility to healthcare services in different regions globally during the COVID-19 pandemic. The review included 21 articles and found that there was a decrease in accessibility to health services, including a decrease in planned surgeries, doctor appointments, patient admission to hospital or emergency rooms and access to medicines during this period, as reported by most studies, though an expansion of online consultations was also noted. Some of the studies included also noted an increase in mortality rate (Tuczyńska, Matthews-Kozanecka, et al.).

The fourth contribution by Sharma et al., conducted a systematic review to consolidate and synthesize the economic evidence of screening programs for cardiovascular diseases (CVD) and diabetes in LMICs. The review included 15 articles and found that numerous innovative screening programs have been piloted. However, based on the available resources and context, the cost-effectiveness may vary for any such program where in only certain circumstances they could be made universal or otherwise targeted just for the high-risk populations (Sharma et al.).

The final piece of this series by Tuczyńska, Staszewski et al., conducted a mini scoping review on the quality of healthcare services available in European countries during the COVID-19 pandemic. The review included 12 articles from studies in the Catalonia, Belgium, France, Germany, Italy, Netherlands, Poland, Sweden, and the United Kingdom. The authors found that patients in the United Kingdom felt that the quality of services was good during the pandemic, though this was not seen in the other regions, where the patients' felt that the quality of care declined. Though the development of telemedicine was considered as a positive impact of the pandemic (Tuczyńska, Staszewski et al.).

We know that investing in women's health improves their rights, reduces gender inequities, while generating health, economic, social, and environmental gains (6, 7). For decades, research for women's health issues has remained grossly underfunded (8). We therefore need more women advocating for, working in women's health research and also in the field of health economics, and this series is helpful step in that direction.

## Author contributions

TS had prepared the manuscript draft while SC has revised it for important intellectual content. Both approved the final version of the manuscript.

## Conflict of interest

The authors declare that the research was conducted in the absence of any commercial or financial relationships that could be construed as a potential conflict of interest.

## Publisher's note

All claims expressed in this article are solely those of the authors and do not necessarily represent those of their affiliated organizations, or those of the publisher, the editors and the reviewers. Any product that may be evaluated in this article, or claim that may be made by its manufacturer, is not guaranteed or endorsed by the publisher.
